# The Role of Musical Attributes in Music-Induced Analgesia: A Preliminary Brief Report

**DOI:** 10.3389/fpsyg.2018.01761

**Published:** 2018-09-26

**Authors:** Krzysztof Basiński, Agata Zdun-Ryżewska, Mikołaj Majkowicz

**Affiliations:** ^1^Department of Quality of Life Research, Faculty of Health Sciences, Medical University of Gdańsk, Gdańsk, Poland; ^2^Faculty of Health Sciences, Pomeranian University in Słupsk, Słupsk, Poland

**Keywords:** pain, music, analgesia, music attributes, music preferences, experimental pain, cold-pressor task

## Abstract

Music-induced analgesia (MIA) is the ability of music to influence pain perception. Although this phenomenon has been extensively studied in recent years, only a few studies have addressed what musical characteristics are optimal for MIA. Here, we present a novel approach to this topic, using a recently proposed model of music attribute preferences. The model addresses three musical dimensions: arousal, valence, and depth. Thirty participants (15 women and 15 men, *M*_age_ = 37.1 years, standard deviation = 15.7) were subjected to experimental pain stimulation (cold-pressor task) while listening to brief music excerpts with characteristics of the three attribute dimensions. Each excerpt was selected to score high on one of the three attributes while being average on the other two, to create three distinct music conditions. There was also a control condition, where participants listened to white noise. Results showed that average pain ratings were significantly lower in the arousal (*p* = 0.002) and depth (*p* = 0.01) conditions compared to the control condition. Furthermore, participants showed increased pain tolerance in musical conditions compared to the control condition (*p* = 0.04). This preliminary report introduces a novel approach to studying MIA in the context of music attribute preferences. With the advent of online music streaming services, this research opens new possibilities for music-based pain interventions.

## Introduction

Modern research into music-induced analgesia (MIA), the ability of music to influence pain perception, began with a seminal work by Gardner et al. (1960), who studied 5,000 patients undergoing dental surgery and found that an intervention involving music and noise reduced pain in 90% of cases. Since then, the topic has been extensively studied with experimental pain stimulation in healthy volunteers (Mitchell et al., 2006; Roy et al., 2008; Zhao and Chen, 2009; Villarreal et al., 2012; Hsieh et al., 2014), and in various clinical settings (Hole et al., 2015). The main mechanisms that have been proposed in MIA are distraction, positive affect, and familiarity (Villarreal et al., 2012). Although substantial research has addressed the impact of music on pain perception, few studies have examined what musical characteristics are optimal for MIA. One study found an analgesic effect of self-chosen preferred vs. non-preferred music in comparison to a silent condition (Hekmat and Hertel, 1993). Another study found that while pain was more tolerable with self-chosen preferred music, decreased perception of pain was observed only in female participants (Mitchell and MacDonald, 2006). Zhao and Chen (2009) found that both happy and sad melodies reduced pain ratings; however, music excerpts were arbitrarily chosen by the experimenters. Knox et al. (2011) performed an acoustical analysis of music that has been rated as highly pain-relieving, finding that the most chosen music expressed contentment.

Here, we present an innovative approach for selecting music for MIA research, which is based on several previous studies on music preferences, understood as individuals’ unique affective reactions to music (Rentfrow et al., 2011, 2012; Greenberg et al., 2015, 2016). We used a model of preferences for musical attributes suggested by Greenberg et al. (2016) that proposes three basic dimensions: arousal, valence, and depth. These dimensions were found using factor analysis, are independent of genre, and are associated with personality. Arousing music is characterized as “intense, forceful, abrasive, or thrilling”; valence refers to “fun, happy, lively, enthusiastic, and joyful”; and depth refers to “intelligent, sophisticated, inspiring, complex, poetic, deep, emotional, and thoughtful” (Greenberg et al., 2016, pp. 3). We sought to determine if musical attribute dimensions contribute distinctly to MIA. If so, future music interventions in pain conditions might focus on music attributes that have the best analgesic properties.

We hypothesized that music rated high on valence would produce a highly positive affective response, thus having a higher analgesic effect than music with a medium valence rating. Similarly, music rated high on depth would be highly cognitively engaging and require more attentional resources to process compared to music with average depth ratings, thus contributing to lower pain perception.

## Materials and Methods

### Participants

Thirty healthy volunteers (15 women and 15 men) aged 20–69 years [*M*_age_ = 37.1 years, standard deviation (SD) = 15.7] were recruited. Participants were asked if they met any of the specific exclusion criteria: diabetes, pain condition, circulatory disorder, hypertension, Raynaud’s disease, previous cold injury, blood clotting problems (Jackson et al., 2005), being pregnant, being a professional musician, or having more than 5 years of formal musical training. Participants had been instructed not to consume any analgesic medication in the 24 h prior to the experiment.

### Stimuli

The musical excerpts were selected from a pool of songs used in previous studies on musical preferences (Rentfrow et al., 2012; Greenberg et al., 2016). These pieces were unknown, commercially unreleased music to ensure that no effects of familiarity would affect the results. For each condition (arousal, valence, and depth), we used a set of eight 15-second excerpts. This was done to provide a wide range of musical pieces during stimulation that were similar in terms of one attribute, but different in terms of genre, instrumentation, and other characteristics. The music for each condition was selected based on factor loadings for each of the excerpts obtained by Greenberg et al. (2016). For every condition, we selected songs that had the highest loadings for the given attribute, while at the same time, having average loadings (±1 *SD*) on other factors. In the control condition the participants listened to white noise. The musical stimuli were delivered at a participant-chosen, comfortable listening level using studio-grade headphones. The volume of the excerpts was normalized.

The pain stimuli were evoked using a cold-pressor test. This test is a widely used, safe, valid, and reliable method of experimental pain induction (Mitchell et al., 2004), and it has been used in previous research on MIA (Mitchell et al., 2006; Finlay and Rogers, 2014). Participants were asked to submerge their non-dominant hand in cold water and to keep it there until it was too uncomfortable to continue. They announced when they started to feel pain. The maximum time of stimulation was set at 2 min. To maintain a stable water temperature and avoid the effect of local heating in the vicinity of the hand, a refrigerated circulatory water bath was used. Based on previous research (Mitchell et al., 2004), the temperature was set to 3°C to provide an average stimulation time of 90–120 s.

### Measures

Pain threshold was measured in seconds as time between the start of the cold-pressor task and the moment when the participant reported the sensation of pain. Pain tolerance was the time until the participant felt too uncomfortable to continue (maximum set at 2 min). After each stimulation, participants were asked to evaluate their pain using an 11-point numerical rating scale (NRS) from 0 (*no pain*) to 10 (*worst possible pain*). Participants rated the intensity of their maximal pain (“when it was the worst”) and average pain (“on average during this trial”). Additionally, participants evaluated the controllability of pain from 0 (*I had it totally under control*) to 10 (*it was totally uncontrollable*). Physiological measures of arousal (blood pressure and heart rate) were taken before the procedure and after each trial.

### Procedure

**FIGURE 1:**
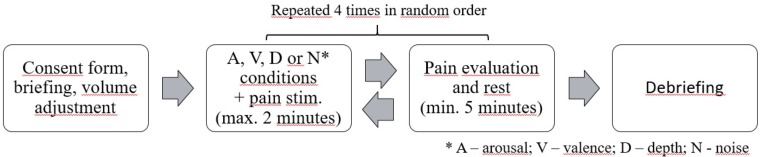
Outline of the experimental procedure.

The procedure followed the recommendations of the Declaration of Helsinki and was approved by the Independent Bioethics Commission for Research of the Medical University of Gdańsk, Poland. Participants were briefed on the procedure, informed of the anonymity of their responses, and that they could withdraw from the experiment at any time. Afterward, they listened to a piece of music unrelated to the actual music stimuli to adjust the playback volume to a comfortable level. Four experimental trials were performed for each participant. The order of the experimental conditions was randomized, as was the order of the musical excerpts. After each pain stimulation, participants immediately placed their hand in a bowl filled with warm water (34–36°C) for a rest period (≥5 min). During the rest period, participants evaluated their maximal and average pain, pain controllability, and their blood pressure and heart rate were measured. After the procedure, participants were debriefed. The procedure overview is presented in **Figure 1**.

### Statistical Analyses

Data were analyzed using R Statistics (R Core Team, 2016) with the packages *dplyr* (Wickham et al., 2017), *Hmisc* (Harrell, 2014), and *ez* (Lawrence, 2016). Plots were created using *ggplot2* (Wickham, 2009) and *cowplot* (Wilke, 2017). The differences in dependent variables between experimental conditions were evaluated using one-way repeated measures analysis of variance (ANOVA) with four factors corresponding to three music conditions and a control condition. All *p*-values were calculated using Greenhouse-Geisser corrections for violations in sphericity. Generalized eta-squared was computed to measure effect sizes. If the ANOVA yielded significant results, *post hoc* comparisons using Bonferroni’s adjustment were made.

## Results

Mean reported average pain intensity throughout the entire 120 trials was 4.92 on an 11-point NRS (*SD* = 2.05), mean reported maximum pain was 6.50 (*SD* = 2.40), and mean perceived controllability was 3.89 (*SD* = 2.76). Average pain threshold was 25.76 s (*SD* = 27.53) and average pain tolerance was 67.12 s (*SD* = 47.15).

**FIGURE 2:**
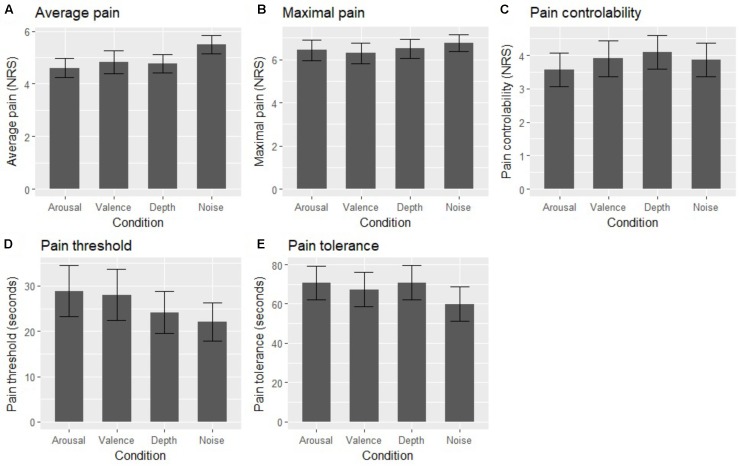
Mean values of average pain **(A)**, maximal pain **(B)**, pain controllability **(C)**, pain threshold **(D)**, and pain tolerance **(E)** in four experimental conditions. Error bars represent 95% confidence intervals for the means.

Significant differences between experimental conditions were observed for average pain ratings [*F*(3,87) = 5.59, *p* = 0.002, η^2^ = 0.028]. Highest ratings were found for the noise condition (*M* = 5.50, *SD* = 1.96), with lower values for music conditions: valence (*M* = 4.83, *SD* = 2.34), depth (*M* = 4.77, *SD* = 1.91), and arousal (*M* = 4.60, *SD* = 1.98). *Post hoc* comparisons showed significant differences between the noise and arousal conditions (*p* = 0.002), as well as between noise and depth (*p* = 0.01). Additionally, significant differences were observed in the measure of pain tolerance [*F*(3,87) = 2.94, *p* = 0.04, η^2^ = 0.008]. Participants had the lowest tolerance in the noise condition (*M* = 59.93, *SD* = 47.74), followed by valence (*M* = 67.33, *SD* = 47.69), arousal (*M* = 70.54, *SD* = 47.00), and depth (*M* = 70.70, *SD* = 47.78). *Post hoc* comparisons did not show differences between any specific pairs of means. No significant differences were observed for maximum pain [*F*(3,87) = 1.04, *p* = 0.37, η^2^ = 0.005], pain controllability [*F*(3,87) = 0.79, *p* = 0.48, η^2^ = 0.005], pain threshold [*F*(3,87) = 1.531, *p* = 0.22, η^2^ = 0.010], or the physiological measures: systolic blood pressure [*F*(3,87) = 1.04, *p* = 0.36, η^2^ = 0.005], diastolic blood pressure [*F*(3,87) = 1.03, *p* = 0.38, η^2^ = 0.008], and heart rate [*F*(3,87) = 0.93, *p* = 0.41, η^2^ = 0.004] (**Figure 2**).

## Discussion

As hypothesized, participants rated their pain significantly lower in the depth condition compared to the control condition. This can be explained by the need to allocate more attentional resources to process more complex, “deep” music, thus providing a distraction from pain. Contrary to our hypothesis, no significant differences were found between the valence and control conditions. This is surprising, as previous studies revealed an analgesic effect only in emotionally pleasant music (Roy et al., 2008). It is possible that music in the valence condition, though happy and positive, did not produce a complex emotional response, perhaps due to the short duration of the musical excerpts. Future research should focus on determining the actual change in affect due to music listening and how this change affects MIA.

Arousing music contributed to significantly lower pain ratings. This may suggest that pain perception is modulated more by emotional arousal rather than positive emotional valence. Another interpretation is that highly arousing music engages more attentional resources, leading to effective distraction from pain, as suggested in other studies (Villarreal et al., 2012). Some participants may have also felt more engaged by arousing music in a generally monotonous experimental setting.

One limitation was the lack of control for participants’ actual preferences for musical attributes. Individual affective reactions to music may be effective predictors of MIA. A larger sample size is needed, as this involves probing for individual differences in music preferences. This work is currently under way. Another limitation is the very broad age range of the participants in the current study, as younger participants may rate cold-pressor pain higher than older ones (Lue et al., 2018). The larger sample size will allow us to look at age as a potential moderator of MIA. Other limitations include only using music from one end of the musical attribute dimensions. Perhaps music rated low on arousal, valence, or depth would produce distinct analgesic effects. Other characteristics of music apart from the attributes studied here may also contribute to MIA (for example relaxation and familiarity). Finally, future research should employ other types of experimental pain-inducing procedures (thermal, pressure, electric, etc.).

Our results suggest that the presented methodology may be useful in determining the music best suited for MIA. This is relevant when developing interventions for non-pharmacological treatment of chronic pain, which is one of the leading causes of disability globally (Hoy et al., 2010). Music-based interventions can be inexpensive, safe, easy to distribute, and effective, especially with the advent of online music streaming services. The approach presented in this work may lead to new algorithms for selecting music best suited for pain management.

## Data Availability Statement

The datasets for this study, as well as scripts used for analysis and technical details of the performed experiment can be found in the GitHub repository: https://k-basinski.github.io/mia_music_prefs/.

## Author Contributions

KB and MM conceived and designed the study. KB and AZ-R performed the study and organized the dataset. KB performed the statistical analysis and wrote the first draft of the manuscript. KB, AZ-R, and MM wrote sections of the manuscript. All authors contributed to manuscript revision, and read and approved the submitted version.

## Conflict of Interest Statement

The authors declare that the research was conducted in the absence of any commercial or financial relationships that could be construed as a potential conflict of interest.
